# Prospective Studies Comparing Structured vs Nonstructured Diagnostic Protocol Evaluations Among Patients With Fever of Unknown Origin: A Systematic Review and Meta-analysis

**DOI:** 10.1001/jamanetworkopen.2022.15000

**Published:** 2022-06-02

**Authors:** William F. Wright, Joshua F. Betz, Paul G. Auwaerter

**Affiliations:** 1 Division of Infectious Diseases, Department of Medicine, Johns Hopkins University School of Medicine, Baltimore, Maryland; 2 Department of Biostatistics, Johns Hopkins Bloomberg School of Public Health, Baltimore, Maryland; 3 The Sherrilyn and Ken Fisher Center for Environmental Infectious Diseases, Johns Hopkins University School of Medicine, Baltimore, Maryland

## Abstract

**Importance:**

Patients meeting the criteria for fever of unknown origin (FUO) can be evaluated with structured or nonstructured approaches, but the optimal diagnostic method is unresolved.

**Objective:**

To analyze differences in diagnostic outcomes among patients undergoing structured or nonstructured diagnostic methods applied to prospective clinical studies.

**Data Sources:**

PubMed, Embase, Scopus, and Web of Science databases with librarian-generated query strings for *FUO, PUO, fever or pyrexia of unknown origin, clinical trial,* and *prospective studies* identified from January 1, 1997, to March 31, 2021.

**Study Selection:**

Prospective studies meeting any adult FUO definition were included. Articles were excluded if patients did not precisely fit any existing adult FUO definition or studies were not classified as prospective.

**Data Extraction and Synthesis:**

Abstracted data included years of publication and study period, country, setting (eg, university vs community hospital), defining criteria and category outcome, structured or nonstructured diagnostic protocol evaluation, sex, temperature threshold and measurement, duration of fever and hospitalization before final diagnoses, and contribution of potential diagnostic clues, biochemical and immunological serologic studies, microbiology cultures, histologic analysis, and imaging studies. Structured protocols compared with nonstructured diagnostic methods were analyzed using regression models.

**Main Outcomes and Measures:**

Overall diagnostic yield was the primary outcome.

**Results:**

Among the 19 prospective trials with 2627 unique patients included in the analysis (range of patient ages, 10-94 years; 21.0%-55.3% female), diagnoses among FUO series varied across and within World Health Organization (WHO) geographic regions. Use of a structured diagnostic protocol was not significantly associated with higher odds of yielding a diagnosis compared with nonstructured protocols in aggregate (odds ratio [OR], 0.98; 95% CI, 0.65-1.49) or between Western Europe (Belgium, France, the Netherlands, and Spain) (OR, 0.95; 95% CI, 0.49-1.86) and Eastern Europe (Turkey and Romania) (OR, 0.83; 95% CI, 0.41-1.69). Despite the limited number of studies in some regions, analyses based on the 6 WHO geographic areas found differences in the diagnostic yield. Western European studies had the lowest percentage of achieving a diagnosis. Southeast Asia led with infections at 49.0%. Noninfectious inflammatory conditions were most prevalent in the Western Pacific region (34.0%), whereas the Eastern Mediterranean region had the highest proportion of oncologic explanations (24.0%).

**Conclusions and Relevance:**

In this systematic review and meta-analysis, diagnostic yield varied among WHO regions. Available evidence from prospective studies did not support that structured diagnostic protocols had a significantly better rate of achieving a diagnosis than nonstructured protocols. Clinicians worldwide should incorporate geographical disease prevalence in their evaluation of patients with FUO.

## Key Points

**Question:**

For patients with fever of unknown origin (FUO) syndrome, is an evaluation using a structured diagnostic protocol vs a nonstructured method associated with an improved diagnostic yield?

**Findings:**

In this systematic review and meta-analysis of 19 prospective trials with individual data from 2627 patients, there was no significant difference in the diagnostic yield between those evaluated through a structured compared with a nonstructured protocol. Analysis by geographic region varied in diagnostic yields, despite the limited number of studies for some locations, and geographic disease prevalence appears to be associated with the outcomes of FUO investigations.

**Meaning:**

These findings suggest that insufficient evidence supports the superiority of structured diagnostic protocols; physicians need to consider geographic disease prevalence when evaluating FUO.

## Teaser

This systematic review and meta-analysis compares diagnostic yield studies using a structured or a nonstructured diagnostic approach to treating patients with fever of unknown origin syndrome.

## Introduction

Although fever of unknown origin (FUO) syndrome is a commonly accepted medical diagnosis, no universally accepted method of postdiagnostic evaluation exists. In 1997, a prospective study^1,2,3^ advised using a qualitative standard definition and structured diagnostic protocol because of limited high-quality evidence supporting the use of prior methods.^4,5^ Most published studies are prospective or retrospective single-center case series that lack a uniform process.^6,7^ Although ample expert opinion exists,^1,2,3,7,8^ substantial missing data in these studies preclude definitive conclusions about an optimal investigative process.

Two retrospective trials from Japan^9^ and Italy^10^ that included 9830 hospitalized patients reported the prevalence of this syndrome ranged from 2.0% to 2.9% based on admission diagnosis. Articles cataloging associated conditions appeared as early as 1930 with insufficient definitions and follow-up.^11^ Petersdorf and Beeson^4^ were the first to prospectively adopt formal criteria by which patients would be classified. Subsequent investigators^5,6,7,8^ modified these criteria to reflect practice changes in medicine and increased outpatient evaluation of these patients, including a recent set of standardized proposed defining criteria.^9^

Petersdorf and Beeson^4^ emphasized patient uniqueness where they should not be “subjected to routine battery of laboratory tests” or structured diagnostic protocols. In contrast, de Kleijn et al^1,2,3^ and Bleeker-Rovers et al^6^ advocate for staged structured diagnostic protocols, particularly when potential diagnostic clues from history, examination findings, or existing diagnostic data do not strongly point to an answer. However, many recent series include undiagnosed cases among patients with FUO, ranging from 8.5% to 51.0% despite intensive evaluations.^7^ Factors influencing outcomes might include differences in defining criteria applied,^1,2,3,5,6,7,11^ minimum diagnostic protocol use, location, and prospective or retrospective study design.^7^

To our knowledge, a statistical comparison between studies using either a structured or a nonstructured diagnostic approach is lacking. We performed a systematic literature review with meta-analysis to explore the hypothesis that a structured compared with a nonstructured diagnostic protocol for patients offers an increased diagnostic yield.

## Methods

### Literature Search

This study protocol follows the guidelines of Preferred Reporting Items for Systematic Reviews and Meta-analyses (PRISMA) Checklist. The present study was exempted from obtaining formal institutional review board approval and the requirement to obtain informed patient consent because it is secondary research of publicly available data.

A search of all prospective clinical studies from January 1, 1997, to March 31, 2021, was executed using PubMed, Embase, Scopus, and Web of Science databases with terms constructed by a university librarian that included the query strings of [*FUO*], [*fever of unknown origin* (Medical Subject Headings [MeSH])], [*PUO*], [*pyrexia of unknown origin* (MeSH)], [*clinical trial*], [*clinical trial* (publication type)], and [*prospective studies* (MeSH)]. English and non-English languages articles were included. The search period was chosen based on the adult FUO criteria by de Kleijn et al in 1997.^1,2,3^ Relevant non-English abstracts or full-text articles were translated using Google Translate. Articles resulting from these searches and relevant cited references were reviewed. Patients meeting any definition of adult FUO^1,2,4,5,8^ were included to minimize the chance of unintended selection bias because there remains no single accepted adult FUO definition. Articles were excluded if patients did not precisely fit any existing adult FUO definition^8^ or studies were not classified as prospective.

Abstracted data included years of publication and study period, country, setting (eg, university vs community hospital), FUO defining criteria, structured or nonstructured diagnostic protocol evaluation, standard FUO category outcomes (eg, infectious diseases, noninfectious inflammatory disorder, oncology, miscellaneous, and undiagnosed), sex, temperature threshold and method of measurement, duration of fever and hospitalization before final diagnoses, and contribution of potential diagnostic clues, biochemical and immunological serologic studies, microbiology cultures, histologic analysis, and imaging studies. Studies were classified according to geographic distribution based on the 6 World Health Organization (WHO) regions.^12^ The United Nations Human Development Index ranking,^13^ years of life expectancy at birth (LEB), and gross national income per capita (GNI) were also included for each representative country.

### Statistical Analysis

Similarities in the frequency of diagnostic outcomes across studies were explored using agglomerative hierarchical clustering based on the Pearson correlation distance.^14^ Diagnostic yield for each study was separately quantified using binomial confidence intervals with frequentist (Agresti-Coull confidence intervals and Wilson score intervals) and bayesian credible (Jeffreys highest posterior density) intervals. Jeffreys prior distribution is a minimally informative prior. Wilson score and Agresti-Coull confidence intervals are established methods for binary proportions.^14^ Both overcome well-known limitations of Wald approximations and are not as conservative as the Clopper-Pearson method.

Logistic regression using generalized linear mixed-effects models were used to explore the variation in diagnostic yield across studies by modeling the odds of a case resulting in a diagnosis according to the type of diagnostic protocol used (structured vs nonstructured), WHO study region,^12^ and study-specific variation. Study-specific random effects were used to quantify and account for variation across studies after accounting for study region and protocol type. A mean population effect on the odds ratio (OR) scale that appropriately accounts for between-study variability was obtained by marginalizing (ie, integrating) over the study-specific effects. Analyses were conducted in aggregate, and stratified results were presented for WHO study regions^12^ in which at least 2 studies of each protocol type (structured and nonstructured) were conducted.

Generalized linear mixed-effects models were fit using the lme4 package, version 1.1-27-1 and GLMMadaptive package, version 0.8-2 (R Foundation for Statistical Computing). Data checking, model fitting and criticism, and graphics were conducted using R, version 4.0.5 (R Foundation for Statistical Computing). Statistical significance was set at 2-sided α = .05.

### Risk of Bias

No methods were used to assess the risk of bias due to missing results in synthesis. The outcome and covariates were complete in all cases. Other types of analyses (eg, a funnel plot) are only applicable to the aggregation of studies where both treatments are applied in the same study.

## Results

### Literature Review

**Figure 1.**
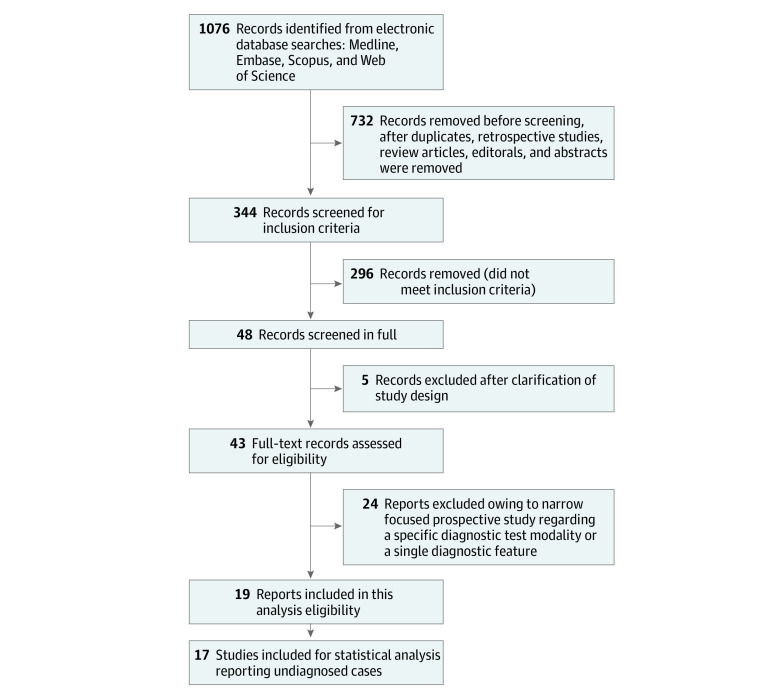
Flow Diagram of Included Studies

The search produced 19 prospective studies^1,2,6,15,16,17,18,19,20,21,22,23,24,25,26,27,28,29,30,31^ (including 2 references for the study by de Kleijn et al^1,2^) meeting the inclusion criteria (Figure 1). Each study’s characteristics and diagnostic yield were considered individually (Table 1). Seventeen studies^1,2,15,16,17,18,19,20,21,22,23,24,25,26,27,28,29,30^ were conducted within a university hospital system, 1 study^6^ within a mixed university and community hospital setting, and 1 study^31^ within a community hospital setting.

**Table 1.** General Characteristics of Studies by the 6 WHO Regional Groupings and 2020 HDI Ranking^a^

Source	No. of participants (% female)	Protocol (yes or no)	Age, median (range), y	HDI rank, No.^b^	GNI per capita, $	LEB, y	FUO criteria	Etiology, No. (%)				
								INF	NIID	ONC	MIS	UD
**Southeast Asian region**												
Kejariwal et al,^16^ 2001	100 (41.0)	No	32 (12-65)	131	6681	69.7	Petersdorf and Beeson^4^	53 (53.0)	11 (11.0)	17 (17.0)	5 (5.0)	14 (14.0)
Bandyopadhyay et al,^24^ 2011	164 (50.0)	No	42 (27-57)	131	6681	69.7	Durack and Street^5^	90 (54.9)	18 (11.0)	36 (21.9)	0	20 (12.2)
Mir et al,^25^ 2014	91 (21.0)	No	NL (16-49)	131	6681	69.7	Petersdorf and Beeson^4^; Durack and Street^5^	40 (43.9)	11 (12.1)	11 (12.1)	4 (4.4)	25 (27.5)
Pannu et al,^30^ 2021	112 (NL)	Yes	NL	131	6681	69.7	Petersdorf and Beeson^4^	48 (42.8)	21 (18.7)	28 (25.0)	3 (2.7)	12 (10.7)
Total	467	NAD	NAD	NAD	NAD	NAD	NAD	231 (49.5)	61 (13.1)	92 (19.7)	12 (2.6)	71 (15.2)
**European region **												
Vanderschueren et al,^18^ 2003	290 (43.0)	No	54 (33-65)	14	52 085	81.6	Petersdorf and Beeson^4^	57 (19.7)	68 (23.4)	29 (10.0)	38 (13.1)	98 (33.8)
De Kleijn et al,^1,2^ 1997	167 (52.0)	Yes	53 (16-87)	8	57 707	82.3	Petersdorf and Beeson^4^	43 (25.7)	40 (23.9)	21 (12.6)	13 (7.8)	50 (29.9)
Ergönül et al,^20^ 2005	80 (51.0)	No	44 (29-59)	54	27 701	77.7	Petersdorf and Beeson^4^; Durack and Street^5^	42 (52.5)	10 (12.5)	14 (17.5)	5 (6.3)	9 (11.3)
Altiparmak et al,^15^ 2001	50 (64.0)	Yes	38 (15-75)	54	27 701	77.7	Petersdorf and Beeson^4^	22 (44.0)	3 (6.0)	13 (26.0)	8 (16.0)	4 (8.0)
Saltoglu et al,^19^ 2004	87 (29.9)	No	38 (14-80)	54	27 701	77.7	Petersdorf and Beeson^4^	51 (58.6)	16 (18.4)	12 (13.8)	2 (2.3)	6 (6.9)
Baicus et al,^17^ 2003	164 (51.8)	Yes	46 (18-78)	49	29 497	76.1	Petersdorf and Beeson^4^	74 (45.1)	30 (18.3)	41 (25.0)	7 (4.3)	12 (7.3)
Robine et al,^26^ 2014	103 (48.0)	No	53 (19-84)	26	47 173	82.7	Durack and Street^5^	12 (11.7)	31 (30.1)	3 (2.9)	5 (4.9)	52 (50.5)
Bleeker-Rovers et al,^6^ 2007	73 (54.8)	Yes	54 (26-87)	8	57 707	82.3	Petersdorf and Beeson^4^	12 (16.4)	16 (21.9)	5 (6.8)	3 (4.1)	37 (50.7)
Kucukardali et al,^21^ 2008	154 (46.1)	Yes	42 (17-75)	54	27 701	77.7	Durack and Street^5^	53 (34.4)	47 (30.5)	22 (14.3)	8 (5.2)	24 (15.6)
Torné Cachot et al,^31^ 2021	87 (47.2)	No	56 (37-75)	25	40 975	83.6	Durack and Street^5^	15 (17.2)	19 (21.8)	13 (14.9)	14 (16.1)	26 (29.9)
Total	1255	NAD	14-87	NAD	NAD	NAD	NAD	381 (30.3)	280 (22.3)	173 (13.8)	103 (8.2)	318 (25.3)
**Eastern Mediterranean region **												
Adil Khalil et al,^22^ 2010	55 (50.9)	Yes	43 (10-76)	123	10 801	70.6	Durack and Street^5^	18 (32.7)	14 (25.5)	9 (16.4)	3 (5.5)	11 (20.0)
Ali-Eldin et al,^23^ 2011	93 (51.6)	No	34 (15-65)	116	11 466	72.0	Petersdorf and Beeson^4^; Durack and Street^5^	39 (41.9)	14 (15.1)	28 (30.1)	0	12 (12.9)
Total	148	NAD	10-76	NAD	NAD	NAD	NAD	57 (38.5)	28 (18.9)	37 (25.0)	3 (2.0)	23 (15.5)
**Western Pacific region **												
Wu et al,^27^ 2018	431 (44.8)	No	NL	85	16 057	76.9	Durack and Street^5^	241 (55.9)	93 (21.6)	62 (14.4)	35 (8.1)	NL
Naito et al,^28^ 2019	141 (55.3)	No	62 (22-94)	19	42 932	84.6	Durack and Street^5^	24 (17.0)	48 (34.0)	22 (15.6)	17 (12.1)	30 (21.3)
Xu et al,^29^ 2020	185 (43.8)	No	53 (32-67)	85	16 057	76.9	Petersdorf and Beeson^4^	97 (52.4)	49 (26.5)	14 (7.6)	NL	NL
Total	757	NAD	NAD	NAD	NAD	NAD	NAD	362 (47.8)	190 (25.1)	98 (12.9)	NAD	NAD

Abbreviations: FUO, fever of unknown origin; GNI, gross national income; HDI, Human Development Index; INF, infectious diseases; LEB, life expectancy at birth; MIS, miscellaneous conditions; NAD, no available data; NIID, noninfectious inflammatory disorders; NL, not listed; ONC, oncology/neoplastic conditions; UD, undiagnosed; WHO, World Health Organization.

^a^ For WHO regional groupings, see WHO^12^; for HDI ranking, see United Nations Development Programme.^13^

The total sample consisted of 2627 participants, of whom 11.7% to 58.6% had an infectious diseases etiology, 6.0% to 34.0% had a noninfectious inflammatory disorder, and 2.9% to 30.1% had an underlying oncologic condition. Undiagnosed cases were reported in all but 2 studies^27,29^ and ranged from 6.9% to 50.7%. Nine studies^1,2,6,15,16,17,18,19,29,30^ with 1228 of the 2627 total participants (46.7%) used the criteria of Peterdorf and Beeson^4^ to classify FUO, and 7 studies^21,22,24,26,27,28,31^ combining 1135 participants (43.2%) were classified by the criteria of Durack and Street.^5^ Three studies^20,23,25^ of 264 participants (10.0%) were classified by either the Petersdorf and Beeson^4^ or Durack and Street^5^ criteria. Data pertaining to mortality rates, spontaneous fever resolution, and patients lost to follow-up are reported in the eTable in the Supplement.

Several reports, including 2 by de Kleijn et al,^1,2^ enrolled patients younger than 18 years^15,16,19,21,22,23,25^ if the patients met the adult FUO criteria. Among studies in this analysis, ages ranged from 10 to 94 years, with female participants ranging from 21.0% to 64.0%. The main reason for excluding variables was missing information. Lack of sufficient data meant that analysis of temperature measurement method, duration of fever and hospitalization before a diagnosis, the contribution of potential diagnostic clues, biochemical and immunological serologic studies, microbiology cultures, histologic analysis, and imaging studies could not be performed.

### Statistical Analysis

**Figure 2.**
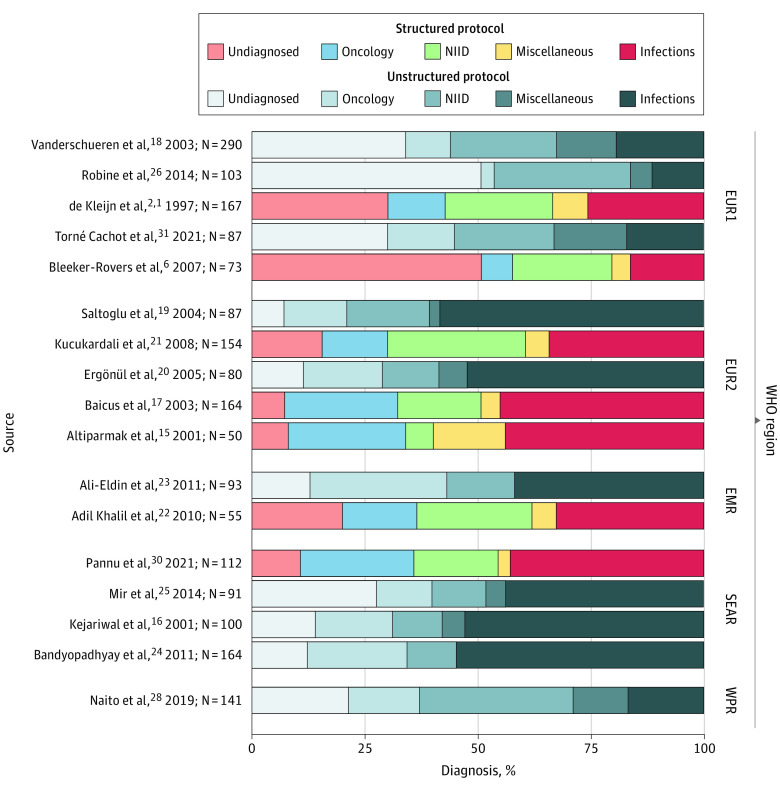
Resulting Diagnoses of Fever of Unknown Origin by Region and Study Among the 19 studies included in the meta-analysis, Wu et al^27^ and Xu et al^29^ are excluded here owing to incomplete reporting of data. EMR indicates Eastern Mediterranean region; EUR1, Belgium, France, the Netherlands, and Spain; EUR2, Turkey and Romania; NIID, noninfectious inflammatory disorders; SEAR, Southeast Asia Region; and WPR, Western Pacific region.

The diagnostic yield (ie, the proportion of cases resulting in a diagnosis) was plotted by study and region (Figure 2) and sorted by structured or nonstructured category (eFigure 1 in the Supplement) for 17 studies,^1,2,6,15,16,17,18,19,20,21,22,23,24,25,26,28,30,31^ because 2 studies did not report undiagnosed cases.^27,29^ Studies with diagnostic yields of greater than 80.0% included 4 with structured diagnostic protocols^15,17,21,30^ and 6 with nonstructured protocols.^16,19,20,23,24,27^ When studies with a diagnostic yield greater than 80.0% are compared, all ranged above the 50th percentile, except China,^26^ which ranked in the fourth percentile on the United Nations Human Development Index ranking list.^13^ Interestingly, the studies with diagnostic yields less than 70.0% all ranked within the top 26 countries economically. When the outcomes were compared by years of LEB and GNI per capita, the countries with the lowest diagnostic yield had LEB ranging from 81.6 to 83.6 years and GNI ranging from $40 975 to $57 707. In contrast, countries with a diagnostic yield greater than 80.0% had LEB ranging from 69.7 to 77.7 years and GNI ranging from $6681 to $29 497 (eFigures 2-4 in the Supplement).

Applying an agglomerative hierarchical clustering algorithm to outcomes data of these 17 prospective studies yielded a dendrogram (eFigure 5 in the Supplement). The WHO region for European studies was divided into 2 categories: EUR2 (Turkey^15,17,19,20,21^ plus Romania^17^) and EUR1 (all other European studies^1,2,6,18,26,31^). There were many studies in the EUR1 region with far more substantial similarities in diagnostic outcomes.

**Figure 3.**
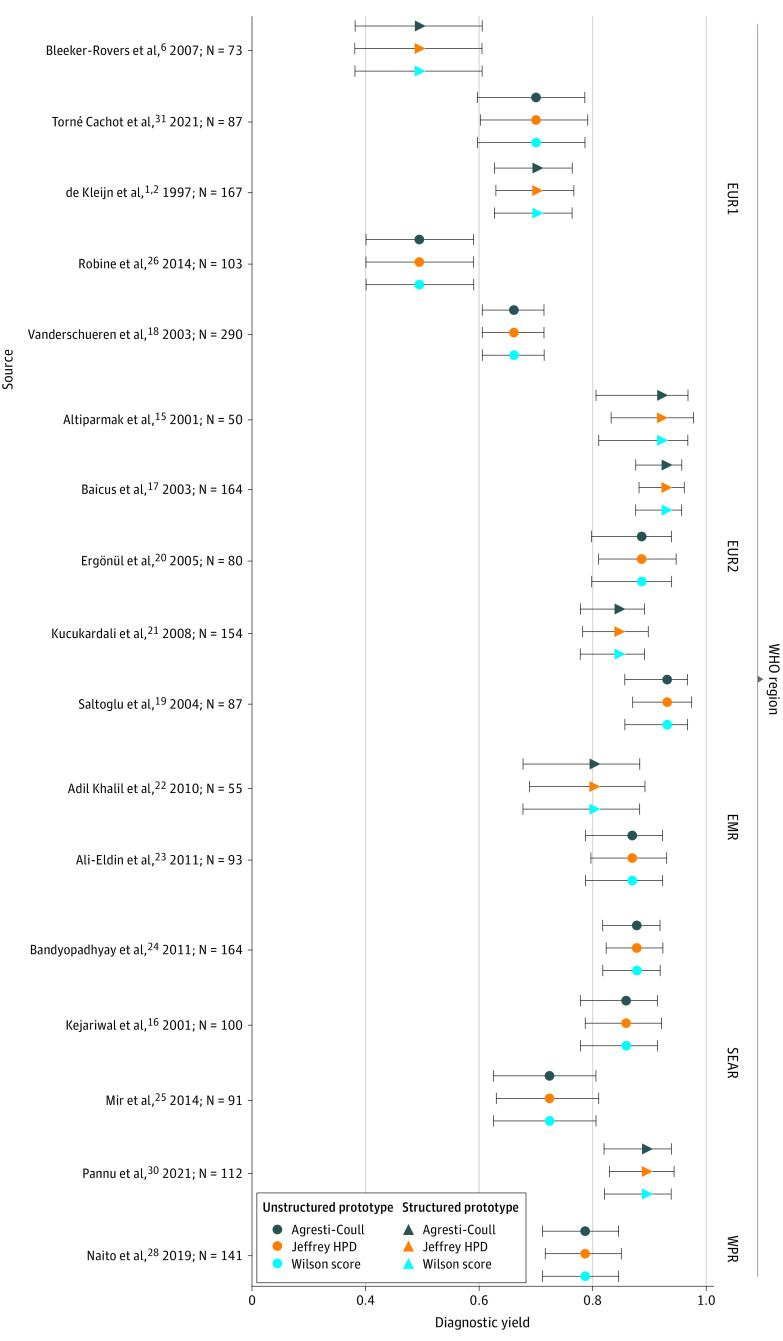
Diagnostic Yield by Study and World Health Organization (WHO) Region Point estimates are given along with 95% uncertainty intervals (Agresti-Coull and Wilson score confidence intervals and Jeffreys bayesian credible interval). EMR indicates Eastern Mediterranean region; EUR1, Belgium, France, the Netherlands, and Spain; EUR2, Turkey and Romania; HPD, highest posterior density; SEAR, Southeast Asia Region; WHO, World Health Organization; and WPR, Western Pacific region.

Western Europe demonstrated a significantly lower diagnostic yield than all other WHO regions in an aggregate model of diagnostic yield (Figure 3). Infection was most prevalent in Southeastern Asia at 49.0%; noninfectious inflammatory disorders, in Western Pacific regions (27.0%); and oncologic explanations, in the Eastern Mediterranean region (24.0%). Variation in study-specific effects demonstrated heterogeneity across studies (SD of study-specific effects on log-OR scale: 0.32; 95% CI, 0.18-0.53). When the mean of this study-specific variation was calculated, negligible associations were observed between protocol type and diagnostic yield: the estimate was small in magnitude, and the data were consistent with a wide range of plausible values for this association (Table 2). The use of a structured diagnostic protocol was not significantly associated with higher odds of yielding a diagnosis than the use of nonstructured protocols (OR, 0.98; 95% CI, 0.64-1.52).

**Table 2.** Summaries of Results From Generalized Linear Mixed-Effects Logistic Regression Models With Study-Specific Coefficients^a^

Model	Term	OR (95% CI)	*P* value
Aggregate	EUR1	1 [Reference]	NA
	EUR2 vs EUR1	5.56 (3.18-9.72)	<.001
	EMR vs EUR1	3.26 (1.64-6.47)	<.001
	SEAR vs EUR1	3.34 (2.08-5.37)	<.001
	WPR vs EUR1	2.23 (1.03-4.82)	.04
	Structured vs unstructured protocol	0.98 (0.65-1.49)	.94
Stratified: EUR1	Structured vs unstructured protocol	0.95 (0.49-1.86)	.88
Stratified: EUR2	Structured vs unstructured protocol	0.83 (0.41-1.69)	.61

Abbreviations: EUR1, Belgium, France, the Netherlands, and Spain; EUR2, Turkey and Romania; NA, not applicable; OR, odds ratio; SEAR, Southeast Asia region; WPR, Western Pacific region.

^a^ Coefficients were obtained by marginalizing or integrating across study-specific effects, allowing interpretation as marginal (population mean) instead of study-specific (conditional) associations. An aggregate model included all studies and diagnostic protocol type (structured vs unstructured) as additive effects. Stratified models were run in each World Health Organization (WHO) region with at least 2 studies of each protocol type (EUR1 and EUR2) to assess the association between protocol and diagnostic yield within WHO regions.

Because this model assumes that the association between diagnostic yield and protocol type is the same worldwide, subsequent modeling stratified by region were fit with at least 2 studies of each protocol type. Stratified results are reported for the EUR1 and EUR2 regions (Table 2). Findings were similar to those of the aggregate model, with estimated small magnitudes of association, and data within each area were consistent with a wide range of plausible ORs. Use of a structured diagnostic protocol was also not significantly associated with higher odds of yielding a diagnosis compared with nonstructured protocols between strata of EUR1 (OR, 0.95; 95% CI, 0.49-1.86) and EUR2 (OR, 0.83; 95% CI, 0.41-1.69).

## Discussion

Our hypothesis that structured protocolized evaluation of FUO would offer a higher diagnostic yield was not borne out through this systematic review–based meta-analysis using prospective studies. To our knowledge, this is the first meta-analysis examining rates of diagnosis between structured compared with nonstructured methods.

The odds of finding a diagnosis were lower in Europe than in other parts of the world. This outcome is consistent with the results reported in a recent systematic review by Fusco et al.^7^ Among 18 prospective and retrospective studies from 2005 to 2015, Fusco et al^7^ also reported a lack of data from the African and American regions, which may have influenced diagnostic outcomes. Similarly, our study was also associated with significant heterogeneity and higher percentages of infectious diseases. Unlike their study,^7^ which reported a lower frequency of oncologic conditions using the Durack and Street criteria,^5^ we had insufficient data to note differences in outcomes based on FUO definitions. However, the estimates derived from studies included in our analyses are more robust owing to the inclusion of only prospective studies from a more extensive set of databases for a greater extended period and different statistical models.

In this meta-analysis, we noted a considerable variation in diagnostic yield across studies, with significant geographic variations in the diagnostic outcomes. Analysis by geographic region based on the 6 WHO regions^12^ was associated with the diagnostic yield, despite the limited number of studies for some locations, suggesting that geographic disease prevalence affects outcomes of FUO investigations. Infection was most prevalent in Southeastern Asia at 49.0%. Noninfectious inflammatory disorders were most common in Western Pacific regions (27.0%). The highest rates for oncologic explanations were in the Eastern Mediterranean region (24.0%).

There was considerable variation in diagnostic yield across WHO regions and studies performed within each region. Plotting the diagnostic yield by United Nations Human Development Index ranking, GNI, and LEB (eFigures 2-4 in the Supplement) resulted in a nonlinear trend. Those at the highest ends of the scale tended to have the worst diagnostic yield, those in the middle had the best diagnostic yield, and those at the bottom end of the scale tended to be somewhere in between. Factors associated with these differences in diagnostic yields in this analysis are unclear. Plausible hypotheses include the following: (1) more rigorous initial investigations leave the most complex cases for evaluation; (2) episodic and benign fevers prompt less rigorous evaluations; (3) conditions yet to be understood exist despite the availability of advanced diagnostic methods and several rounds of evaluations within a higher income environment of clinical experts; (4) diagnostic methods improve over time; (5) variations associated with the ecology of medical care are present^32^; and (6) differences exist within populations that have to do with inflammatory tendencies vs infection prevalence, which appears to be diagnosed more readily.

A question remains: can there be an optimal diagnostic protocol to improve diagnostic yield, and for whom or where would it be most applicable? If this search is based on the diagnostic protocol with the least percentage of undiagnosed cases (eg, highest correlation), only 2 studies^15,17^ used structured protocols reporting rates less than 10.0% despite differing protocols. However, these 2 studies enrolled patients with FUO according to criteria by de Kleijn et al.^1,2^ Therefore, further exploration into the most valuable elements of these 2 protocols might inform an optimal protocol. The positive practical implications of our research results appear to be clear, suggesting physicians worldwide should initially consider geographic disease prevalence when evaluating these patients.

Although this meta-analysis did not support using a structured FUO protocol, that does not exclude additional, more extensive studies exploring optimal diagnostic methods. The goal of FUO research should be to improve the accurate diagnosis and facilitate efficiency with as few tests as required. Possible concepts for future studies include (1) use of a standard contemporary criterion to classify adults with FUO^8^ that would enhance research by allowing better comparison of studies; (2) use of geographic disease epidemiology^8^ and potential diagnostic clues in formulating disease hypotheses, because cases without these clues are particularly challenging to diagnose^2,3,6^; (3) design of initial agnostic panel-based diagnostic testing strategies to meet geographic variations in disease, particularly in patients without potential diagnostic clues; and (4) earlier incorporation of advanced diagnostic methods (eg, 18 F-fludeoxyglucose–positron emission tomography with or without computed tomography or unbiased next-generation sequencing methods).^33,34^ The most definitive approach would be developing a multicenter FUO network that could prospectively evaluate structured vs nonstructured diagnostic investigations incorporating these elements with geographic disease epidemiology.

Finally, undiagnosed disorders are challenging for patients, families, and clinicians. Therefore, forging an FUO network to evaluate patients with undiagnosed diseases that fosters the breadth of expert collaborations and builds on the principles of the precision diagnostic model implemented by the National Institutes of Health Undiagnosed Diseases Network^35^ might reduce undiagnosed FUO cases. By improving testing efficiency and patient outcomes, such a network would leverage findings to facilitate better patient care, including added information about existing diseases as well as the discovery of novel prolonged febrile conditions among the many remaining without diagnoses.

### Limitations

Although this analysis has strengths, it also has notable limitations. First, we did not have access to individual-level data from studies as published data, all differing in reported information. Some geographic regions also lacked data for comparison, such as Africa and North and South America. Second, regression models using aggregated data can only account for what is consistently measured. The statistical adjustment for potential confounding factors is limited by the number of published, prospective studies and the completeness and consistency of their reporting. Only 4 trials, 2 using structured protocols^1,2,6^ and 2 using nonstructured protocols,^28,31^ reported some data regarding the contribution of potential diagnostic clues, making for insufficient comparison. Other variables may explain the considerable variation between studies in the same region, hospital system, or facility. For example, recent travel or immigration status, seasonality, the number of rounds of clinical evaluation performed per patient, the number and type of physicians involved in the evaluation processes, access to medical care, and availability of specialized testing methods. Understanding how the individual patient seeks care within a particular society might offer insight into the persistence of FUO patterns and may reveal disparities between demographic groups or in the use of health care resources (ie, ecology of medical care).^32^ More precise comparisons of diagnostic protocols require controlling for these differences. In addition, it is possible that unmeasured confounding (ie, factors associated both with diagnostic protocol type and yield that are not accounted for in statistical models) introduced bias.

Other possible limitations might include the search terms used, exclusion criteria, and databases. Despite using many databases, including non-English language sources, and exploring some gray literature, such as abstracts and communications, pertinent articles may be missed. Finally, studies with positive findings and better diagnostic performance are more likely to be published than studies with negative results or inferior performance, which would introduce publication bias.

## Conclusions

This meta-analysis of recent prospective study data demonstrated insufficient evidence to support a positive association between using a structured diagnostic protocol and diagnostic yield in cases of FUO syndrome. However, analysis by geographic region did show clear differences in rates and types of diagnoses. This supports the argument that physicians worldwide should incorporate geographic disease prevalence when evaluating these patients. Given the considerable variation across regions and studies, larger-scale research is needed to sufficiently understand the sources and magnitudes of variation in FUO cases. Whether a universal FUO protocol can be developed or enhanced and tailored to geography and widely available resources has yet to be discerned. Clinicians evaluating these patients should remain vigilant for clues based on individual presentations and consequently acknowledge that a nonstructured evaluation protocol might be an appropriate choice for some or most patients.

## Notes

Supplement. **eTable.** Studies Reporting Rates of Mortality, Spontaneous Fever Resolution, and/or Lost to Follow-up **eFigure 1.** Confidence Intervals for the Proportion of Diagnosed Cases by Structured and Nonstructured Study Type **eFigure 2.** Diagnostic Yield of Structured and Nonstructured Studies Plotted Against the United Nations Human Development Index Ranking (HDI) Score **eFigure 3.** Diagnostic Yield of Structured and Nonstructured Studies Plotted Against Gross National Income (GNI) per Capita as Based Upon the United Nations Human Development Index Ranking **eFigure 4.** Diagnostic Yield of Structured and Nonstructured Studies Plotted Against Life Expectancy at Birth (LEB) as Based Upon the United Nations Human Development Index Ranking **eFigure 5.** Dendrogram Illustrates the Similarities in FUO Diagnostic Outcomes Between Studies
