# Supplementary material for: Prospective Studies Comparing Structured vs Nonstructured Diagnostic Protocol Evaluations Among Patients With Fever of Unknown Origin: A Systematic Review and Meta-analysis
Source: JAMA Netw Open. 2022 Jun 2;5(6):e2215000. doi: 10.1001/jamanetworkopen.2022.15000 (PMC9164007; doi:10.1001/jamanetworkopen.2022.15000)
Supplement: Supplement. — eTable. Studies Reporting Rates of Mortality, Spontaneous Fever Resolution, and/or Lost to Follow-up eFigure 1. Confidence Intervals for the Proportion of Diagnosed Cases by Structured and Nonstructured Study Type eFigure 2. Diagnostic Yield of Structured and Nonstructured Studies Plotted Against the United Nations Human Development Index Ranking (HDI) Score eFigure 3. Diagnostic Yield of Structured and Nonstructured Studies Plotted Against Gross National Income (GNI) per Capita as Based Upon the United Nations Human Development Index Ranking eFigure 4. Diagnostic Yield of Structured and Nonstructured Studies Plotted Against Life Expectancy at Birth (LEB) as Based Upon the United Nations Human Development Index Ranking eFigure 5. Dendrogram Illustrates the Similarities in FUO Diagnostic Outcomes Between Studies [file jamanetwopen-e2215000-s001.pdf]

Supplementary Online Content

Wright WF, Betz JF, Auwaerter PG. Prospective studies comparing structured vs nonstructured diagnostic protocol evaluations among patients with fever of unknown origin: a systematic review and meta-analysis. *JAMA Netw Open*. 2022;5(6):e2215000. doi:10.1001/jamanetworkopen.2022.15000

eTable. Studies Reporting Rates of Mortality, Spontaneous Fever Resolution, and/or Lost to Follow-up

eFigure 1. Confidence Intervals for the Proportion of Diagnosed Cases by Structured and Nonstructured Study Type

eFigure 2. Diagnostic Yield of Structured and Nonstructured Studies Plotted Against the United Nations Human Development Index Ranking (HDI) Score

eFigure 3. Diagnostic Yield of Structured and Nonstructured Studies Plotted Against Gross National Income (GNI) per Capita as Based Upon the United Nations Human Development Index Ranking

eFigure 4. Diagnostic Yield of Structured and Nonstructured Studies Plotted Against Life Expectancy at Birth (LEB) as Based Upon the United Nations Human Development Index Ranking

eFigure 5. Dendrogram Illustrates the Similarities in FUO Diagnostic Outcomes Between Studies

This supplementary material has been provided by the authors to give readers additional information about their work.

© 2022 Wright WF et al. *JAMA Network Open*.

eTable. Studies Reporting Rates of Mortality, Spontaneous Fever Resolution, and/or Lost to Follow-up

[Author last name et al], [Citation #], [Year]	Study size (n)	Mortality rate overall (n, %)	Spontaneous fever resolution of *undiagnosed cases (n, %)	Patients lost to follow up (n, %)
**Eastern Mediterranean Region (EMR)**				
Adil Khalil et al [22], 2010	55	4, 7.3	NL	NL
Ali-Eldin et al [23], 2011	93	4, 4.3	5, 41.7	3, 3.2
Total	148			
**European Region (EUR)**				
DeKleijn et al [1, 2], 1997	167	20, 12.0	37, 74.0	NL
Altiparmak et al [15], 2001	50	6, 12.0	2, 50.0	NL
Vanderschueren et al [18], 2003	290	34, 18.0	NL	41, 14.1
Baicus et al [17], 2003	164	NL	9, 75.0	NL
Saltoglu et al [19], 2004	87	11, 12.6	5, 83.3	NL
Bleeker-rovers et al [6], 2007	73	1, 1.4	16, 43.2	NL
Robine et al [26], 2014	103	11, 10.7	20, 38.5	NL
Cachot et al [31], 2021	87	6, 6.9	20, 76.9	NL
Total	1,021			
**South-East Asian Region (SEAR)**				
Kejariwal et al [16], 2001	100	NL	11, 78.6	NL
Mir et al [25], 2014	91	1, 1.1	24, 96.0	NL
Pannu et al [30], 2021	112	NL	12, 63.2	NL
Total	303			

**The denominator for undiagnosed cases is listed in Table 1 of the article.*

Abbreviations: NL, not listed

© 2022 Wright WF et al. *JAMA Network Open*.

eFigure 1. Confidence Intervals for the Proportion of Diagnosed Cases by Structured and Nonstructured Study Type

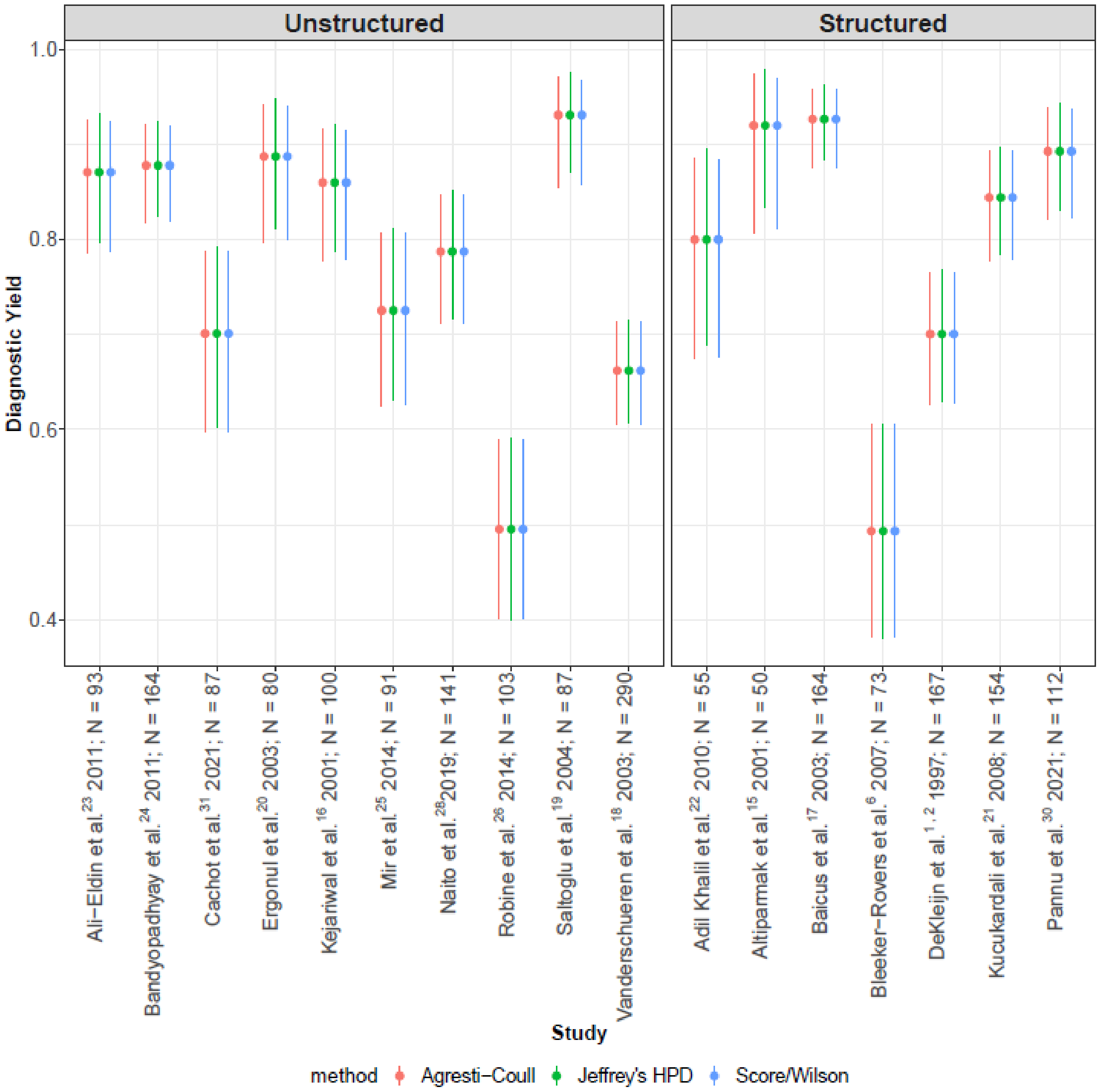

Methods include both frequentist (Agresti-Coull, Wilson), and Bayesian (Jeffreys) confidence intervals.

© 2022 Wright WF et al. *JAMA Network Open*.

eFigure 2. Diagnostic Yield of Structured and Nonstructured Studies Plotted Against the United Nations Human Development Index Ranking (HDI) Score

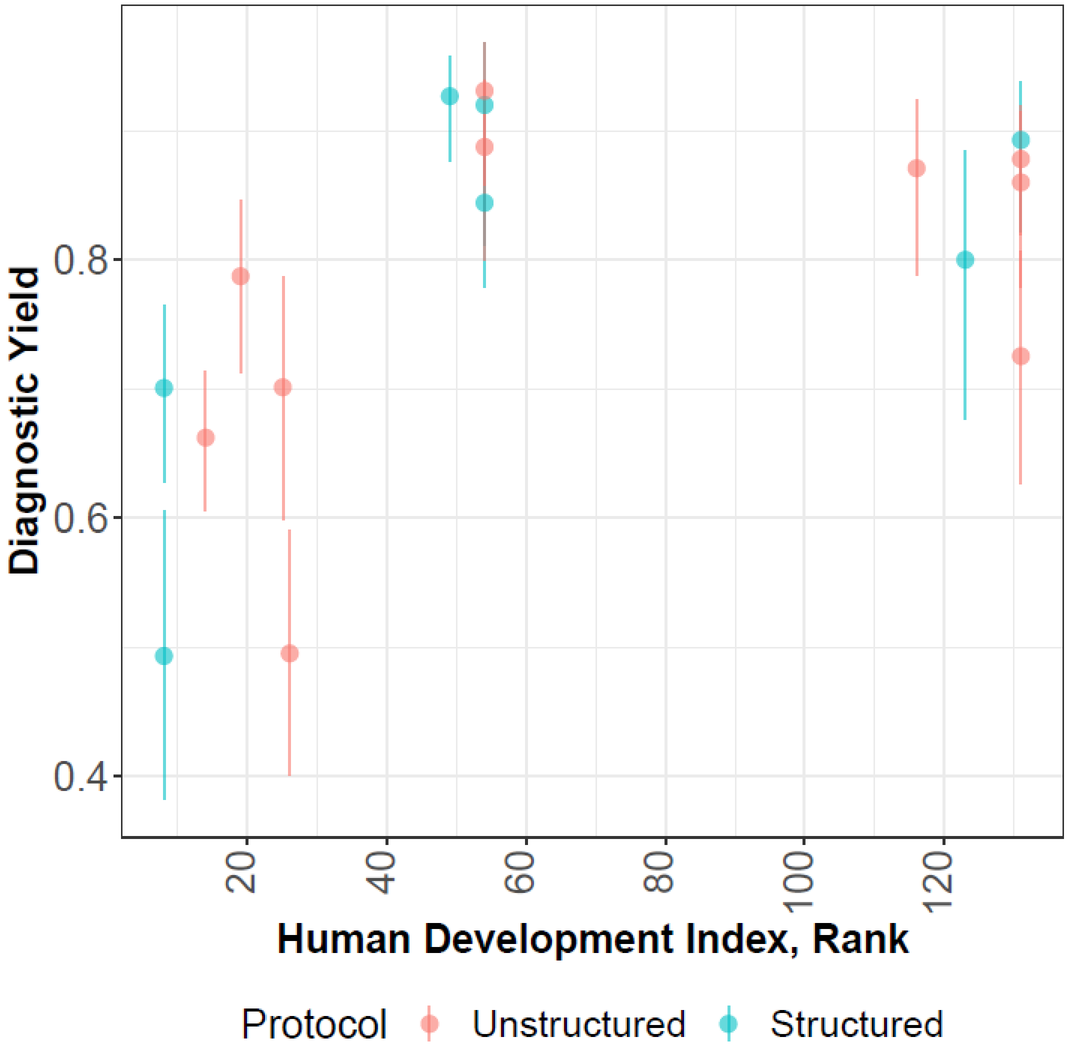

© 2022 Wright WF et al. *JAMA Network Open*.

eFigure 3. Diagnostic Yield of Structured and Nonstructured Studies Plotted Against Gross National Income (GNI) per Capita as Based Upon the United Nations Human Development Index Ranking

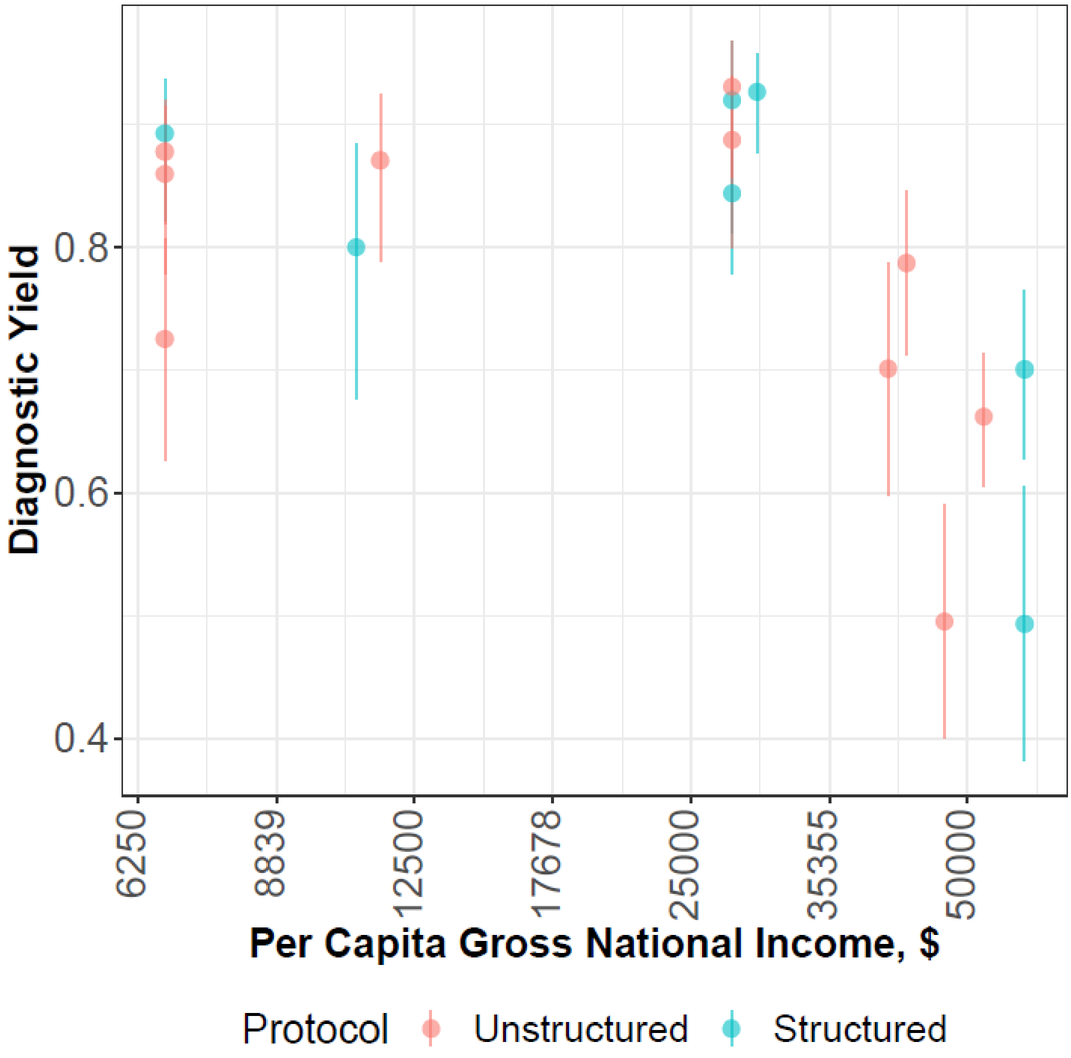

© 2022 Wright WF et al. *JAMA Network Open*.

eFigure 4. Diagnostic Yield of Structured and Nonstructured Studies Plotted Against Life Expectancy at Birth (LEB) as Based Upon the United Nations Human Development Index Ranking

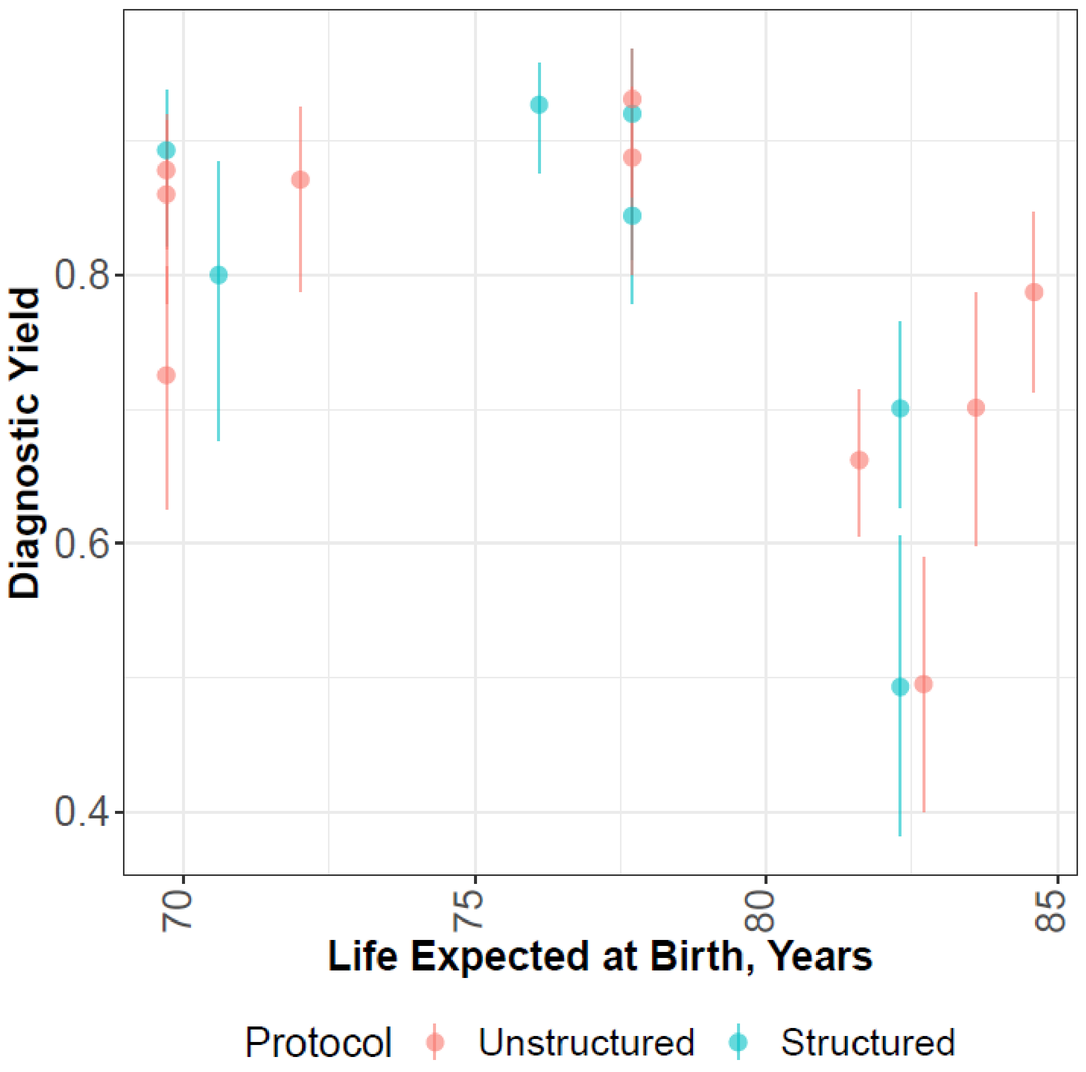

The observation that countries with greater income and longer lifespan is counter intuitive as greater use of medical technologies would assume diagnostic outcomes would be higher.

© 2022 Wright WF et al. *JAMA Network Open*.

eFigure 5. Dendrogram Illustrates the Similarities in FUO Diagnostic Outcomes Between Studies

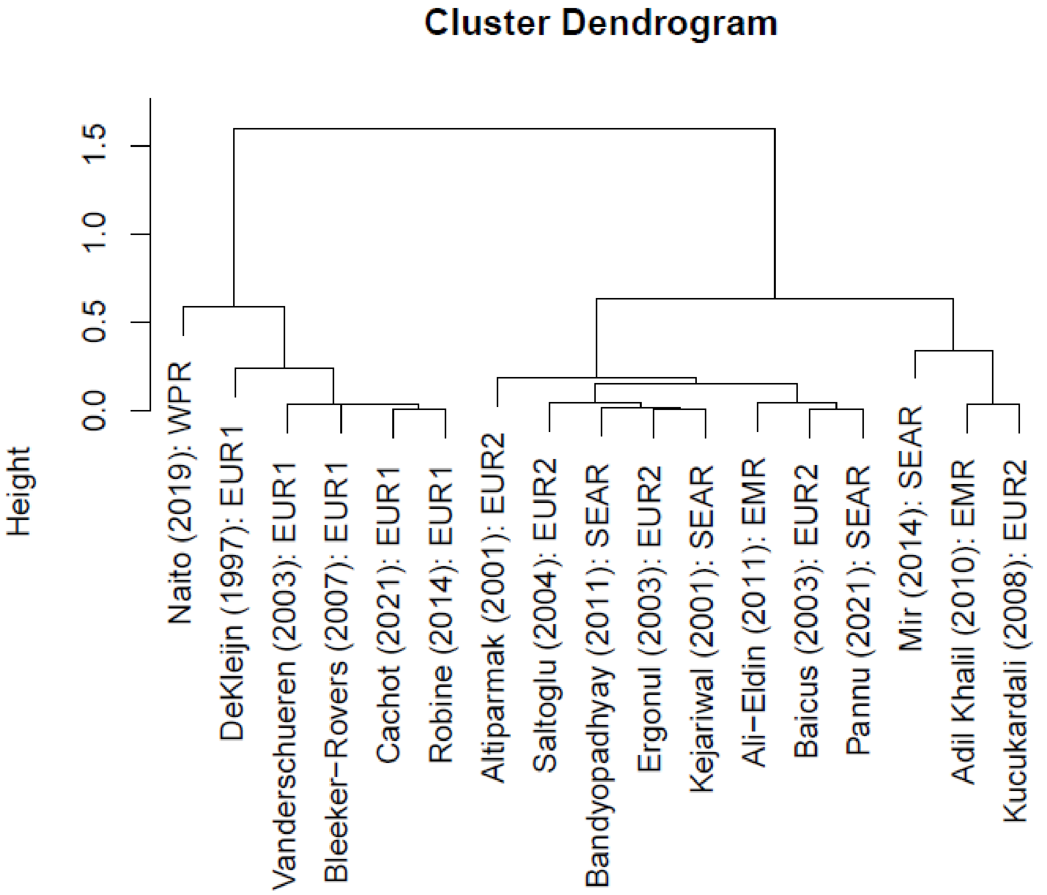

Dissimilarity, measured by a Pearson Correlation distance, is represented by the vertical distance between linkages: the studies with the most similar frequencies of outcomes across diagnostic categories are linked closer to the bottom margin of the plot.

© 2022 Wright WF et al. *JAMA Network Open*.
